# Editorial: HF2Cancer: Exploring bidirectional interaction between cardiovascular diseases and cancer

**DOI:** 10.3389/fcvm.2023.1145780

**Published:** 2023-02-01

**Authors:** Canan G. Nebigil, Michael W. Y. Chan

**Affiliations:** ^1^Regenerative Nanomedicine, UMR 1260, INSERM, University of Strasbourg, Strasbourg, France; ^2^Department of Biomedical Sciences, National Chung Cheng University, Chiayi City, Taiwan

**Keywords:** heart failure, cancer, anti-cancer drugs, population studies, risk factors, biomarkers, genetic variance

## Introduction

Cardiovascular diseases (CVDs) and cancer are responsible for 50% of all deaths in middle-aged people all over the word (1). Indeed, both diseases share common risk factors, including obesity, diabetes, lifestyle, and aging (2). They also exhibit common pathophysiological and genetic mechanisms such as inflammation, neuro-hormonal activation, oxidative stress, clonal hematopoiesis, and a dysfunctional immune system (3). These diseases are tightly linked (4), which is supported by recent epidemiological studies and case control studies, demonstrating that especially heart failure (HF) patients have a higher risk to develop cancer (5–9). Preclinical studies have shown that indeed, HF is an oncogenic stimulus (10–12), promoting tumor growth in distant organs such as the lung and colon. Furthermore, some of the cancer markers can estimate CV mortality and CVD (13).

A better understanding and early diagnosis of CVD and cancer development are critical to delivering timely and targeted prevention strategies and to reduce the world healthcare economic burden. Moreover, clinical awareness is essential to optimize treatment strategies of patients having developed cancer with a history of CVD.

In this Research Topic, we covered the expert reviews on epidemiological studies showing cancer itself leads to a higher risk of death from CVD, the novel nomogram model, some multimodal imaging guides and cardiac biomarkers to detect or predict the risk of CVDs in cancer patients, characterization of possible target molecules to prevent or treat cardiovascular damages induced by the cancer therapy.

Chianca et al. discussed and recapitulate the evidence on cardiovascular and oncological biomarkers in the field of cardio-oncology, focusing on their role in risk stratification, early detection of cardiotoxicity, follow-up, and prognostic assessment. Interestingly, these biomarkers may play key roles to better understand the common pathophysiology of both cancer and HF.

Posch et al. discussed how dynamic cardiotoxicity risk assessment can be achieved by monitoring left ventricular ejection fraction (LVEF), high-sensitive cardiac troponin T (hs-cTnT), and N-terminal pro B-type natriuretic peptide (NT-proBNP) in women with HER2^+^ early breast cancer undergoing primarily trastuzumab-based therapy. They demonstrated that the pre-treatment and longitudinal LVEF trajectory but not hs-cTnT or NT-proBNP can be used for a dynamic assessment of cardiotoxicity risk in this group of patients.

Curtiaud et al. discussed the major etiologies and management of cardiogenic shock related to the acute cardiomyopathy and pulmonary embolism, myocarditis, Takotsubo syndrome, cardiac tamponade, cardiac herniation, neoplastic cardiac infiltration in cancer patient, suggesting an essential multidisciplinary collaboration between intensivists, cardiologists, cardiac surgeons, and oncologists in these critical situations.

In Turk and Kunej's screening of PubMed data base for genetic risk factors in patients with both cancer and CVD, and reviewing of the 181 articles and visualizing the gene-disease network by Cytoscape and the enrichment analysis, they demonstrated that genetic risk factors associated with the comorbidity of cancer and CVDs are significantly enriched in DNA damage repair (DDR) pathways. They suggested requirement of functional studies to elucidate contribution of DDR pathways in the pathophysiology to CVDs.

Guler et al. discussed the preclinical evidences that unravel the molecular pathways and targets in bilateral connection between cardiac injury (HF and early cardiac remodeling) and cancer. They specifically emphasized the cardiac- and cancer-secretoms as possible disease-specific biomarkers and therapeutic targets. They also highlighted the several studies speculating whether cardiovascular drugs are promoter or suppressor of cancer incidence.

Cao et al. showed that Hong Huang Decoction, a Chinese herbal medicine, restores the levels of interleukins such as IL-6, IL-10, superoxide dismutase (SOD), and nitric oxide (NO), as well as pro-inflammatory cytokines such as tumor necrosis factor-α (TNF-α), thereby modulating oxidative stress to protect against antracyline-mediated cardio toxicity in breast cancer patients. They also mentioned that global longitudinal strain is a better detection method of myocardial damage in these patients as compare to LVEF.

Si et al. reported that in a patient with eosinophilic leukemia with progressive eosinophilic myocarditis, multimodality imaging can provide early diagnosis of fibroblast activation, and an anti-fibrotic therapy after heart failure can be a principle early treatment.

Zhang G. et al. summarized the mechanisms of ferroptosis, a new form of regulated cell death that was induced by doxorubicin, in respect to an iron accumulation and metabolism in cardiomyocytes. They also discussed the prevention and treatment of cardiotoxicity targeting ferroptosis.

Wang H. et al. developed and validated a nomogram based on Cox regression to predict the survival of around 20,000 patients with colorectal cancer recruited from 4 centers in China between 2011 and 2017, hoping to improve the prognostic evaluation ability.

Wang W. et al. showed that the signaling pathway that is involved in coiled-coil containing protein kinase (RhoA/ROCK), an inhibitory signal to block regeneration contributes to the inhibiteur de tyrosine kinase anti-VEGFR2, apatinib-mediated hypertension in the gastric cancer, and Y27632 as an inhibitor of RhoA/ROCK has a potential for the prevention and treatment of this type of hypertension.

Murtaza et al. evaluated 18 studies, and found that cancer patients with atrial fibrillation had higher mortality rate and recommended that extra care and specific measures must be taken in the management of cancer patients with new-onset atrial fibrillation.

Rushton et al. demonstrated that increased risk of cardiac event was associated with age (>60) and longer treatment with trastuzumab in HER2^+^ breast cancer patients.

Wu et al. found that the serum adrenomedullin concentration is associated with the incidence of myocardial ischemic T wave change among lung cancer patients, suggesting adrenomedullin as a potentially valuable predictor for heart ischemia in lung cancer patients.

Li J. et al. demonstrated that coronary atherosclerotic heart disease (CAD) is an independent risk factor for cancer, and digestive, respiratory and urogenital cancers are independent risk factors for CAD. Indeed, they also showed that high alanine aminotransferase is correlated with low cancer incident.

Zhang S. et al. reported a 66-year-old woman with syncope caused by carotid sinus syndrome who was eventually diagnosed with advanced nasopharyngeal carcinoma and after diagnosis and treatment, the patient had no recurrence of syncope, suggesting that nasopharyngeal carcinoma is the potential intrinsic causes of syncope.

## Conclusion and future challenges

We think that the Research Topic of *Heart failure (HF) to Cancer (HF2Cancer): Exploring bidirectional interaction between cardiovascular diseases and cancer* provides awareness of new challenges and future directions in the field of cardio-oncology from fundamental scientist to the clinicians. Thus, a better understanding and early diagnosis of HF and cancer development play key roles to delivering timely and targeted prevention strategies to reduce the world healthcare economic burden.

## Author contributions

CN wrote the editorial information. Both CN and MC have approved the submitted version.
